# Risky family environment, white matter organization, and effective parenting in expectant fathers

**DOI:** 10.1017/S0954579425100679

**Published:** 2025-09-22

**Authors:** Sofia I. Cárdenas, Van Truong, Genesis Flores, Fang-Cheng Yeh, Darby E. Saxbe, Vidya Rajagopalan

**Affiliations:** 1 Department of Psychology, University of Southern California, Los Angeles, USA; 2 Department of Pediatrics, Children’s Hospital Los Angeles, Keck School of Medicine, University of Southern California, Los Angeles, USA; 3 Department of Neurological Surgery, University of Pittsburgh, Pittsburgh, USA

**Keywords:** Fatherhood, fractional anisotropy, parenting, risky family environment, transition to parenthood

## Abstract

Fathers have a unique and critical role in children’s development, but limited empirical studies have examined prenatal predictors of fathers’ parenting behaviors. Exposure to early life stressors may alter adult brain white matter fibers, especially in fibers supporting optimal cognitive and emotional functioning. As such, men with experiences of early life stressors, such as risky family environments, may enter parenthood with neurobiological differences that impact their ability to provide optimal parenting. Few studies focus on early life stressors on men’s prenatal neurobiology and subsequent parenting outcomes. This study of first-time fathers (*n* = 41; *M*_age_ = 31.81 years; 32% Hispanic; 32% White; 24% Asian American; 7% Black; 5% Multiracial) investigated whether risky family environments would be associated with prenatal white matter organization and postpartum parenting (infants’ *M*_age_ = 6.96 months). White matter organization was quantified through fractional anisotropy (FA), a measure of the directionality of the fibers within the tissue. Fathers reporting riskier family environments exhibited lower FA in white matter tracts like fornix and cingulum, which support connections between brain areas underlying memory and emotion regulation. Lower FA in these regions predicted less effective parenting postpartum. Findings provide insight into intergenerational transmission of family risk.

## Introduction

Fathers make important contributions to children’s social, cognitive, and linguistic development (Cabrera et al., 2014; Feldman, 2015; Kim & Swain, 2007). Previous evidence indicates that mothers, fathers, and even nonparents show neural activation to infant stimuli in brain areas involved in motivation and reward (Feldman, 2015). This suggests that humans have a common neural circuitry that supports caregiving behavior. Biobehavioral models of fatherhood have sought to characterize contextual factors that may affect the parenting relationship (Alyousefi-van Dijk et al., 2021; Bakermans-Kranenburg et al., 2019; Saxbe et al., 2018). However, current models have not fully considered risk factors, such as fathers’ stressful early experiences within their families of origin, that may increase the risk of suboptimal infant–father relationships. This is concerning, given the extensive research that stressful early life experiences impact men’s biology and behavior distinctly from that of females (E. M. Condon et al., 2022). Furthermore, given the prevalence of stressful early life experiences, researchers have begun to conceptualize child development through “two-generation” frameworks that seek to link and promote parent and child mental health (Fisher et al., 2016; Stoltenborgh et al., 2015). In line with two-generation frameworks, greater empirical research is needed to examine the role of men’s stressful early life experiences on their neurobiology during the transition to parenthood (Cardenas et al., 2022).

Being raised in an environment marked by stress has been linked with neurodevelopmental differences in brain structure. Specifically, studies of adults with histories of adverse childhood experiences show altered brain structure in many areas, such as in the hippocampus and prefrontal cortex, as well as functional changes in the amygdala (Bremner, 2002, 2003, 2006; Dannlowski et al., 2012; Teicher et al., 2012). Emerging evidence also shows a link between early life stress and white matter (WM), which comprises a network of neural fibers of axons covered in myelin that facilitates efficient communication between regions in the brain (Deoni et al., 2011; Nakama et al., 2023). For instance, both adults with post-traumatic stress disorder (PTSD) and childhood trauma exposure, as well as children diagnosed with PTSD, have lower WM connectivity in the corpus callosum, the largest and most expansive WM tract (Siehl et al., 2018). Additionally, chronic stress is linked with lower WM connectivity in the cingulum, a tract that connects the limbic lobe and the cingulate gyrus, in both clinical and subclinical populations (Zhang et al., 2013). Thus, changes to WM connectivity due to early life stress are likely to impact human development across the lifespan.

Life stressors continue to shape the brain beyond early childhood (Hyde et al., 2022). Though WM develops rapidly over the first five years of life (Barnea-Goraly et al., 2005; Fields, 2008), children continue to experience dynamic changes in the brain, including WM, into early adolescence (Corrigan et al., 2021). During periods of plasticity, children’s brains may be particularly vulnerable to stressors (Andersen & Teicher, 2008; Hyde et al., 2022; Tomoda et al., 2024; Tottenham & Galván, 2016; Tottenham & Sheridan, 2009). As a result, more research is needed to examine the biobehavioral impact of life stressors beyond early childhood.

Fractional anisotropy (FA) is one specific measure of WM connectivity that may provide insight into how stressful early experiences shape parent neurobiology. FA, a scalar value that quantifies the degree of diffusion directionality or anisotropy within WM fibers, is derived by diffusion-weighted imaging (DWI). DWI noninvasively measures water molecules’ diffusion within WM fibers (Alexander et al., 2007; Du et al., 2021). Therefore, FA is a biological marker of WM organization (Alexander et al., 2007). Lower FA has been associated with poor outcomes, such as psychopathology, in adult individuals with a history of abuse as well as adolescents with a history of child abuse (Choi et al., 2009; Huang et al., 2012). However, few studies examining the impact of early life stress on subsequent parenting have incorporated measures of FA.

Few empirical studies have examined the impact of early life stress on fathers’ brain structure and parenting. However, neuroimaging studies have found sex differences in the effects of early life stress on brain function and structure (E. M. Condon et al., 2022). For example, a functional study revealed that males, but not females, with a history of neglect showed larger right amygdala volumes, which mediated the relationship between early life stress and later anxiety symptoms (Roth et al., 2018). Another study found that males, but not females, reporting maltreatment histories showed altered WM connectivity in corticolimbic tracts connecting the prefrontal and limbic brain areas (e.g., cingulum, uncinate fasciculus, (Ugwu et al., 2015)). This suggests that early life stress may yield sex-specific effects on patterns of WM connectivity in areas of the brain supporting emotion regulation. Furthermore, many of these functional and structural differences occur in brain regions relevant to parenting behavior (J. T. Condon et al., 2022). Existing biobehavioral theories of fathers’ caregiving behavior emphasize the importance of executive functions (e.g., emotion regulation, planning, multitasking) for optimal caregiving (Feldman et al., 2019). Thus, early life stress may impact men’s caregiving behavior by shaping corticolimbic circuitry critical for executive functioning.

Only one study has directly examined the relationship between early life stress, WM organization, and parenting in expectant and new fathers (Alyousefi-van Dijk et al., 2021). This study found that fathers’ experiences of childhood maltreatment were associated with the ability to modulate handgrip responses, a proxy for self-regulation, while listening to infant cry sounds. While childhood maltreatment experiences did not predict WM organization, greater WM organization in the uncinate fasciculus (UF) moderated the association between early-life stress and handgrip responses during infant cry sounds. More specifically, expectant and new fathers with higher FA in the UF had a weaker association between childhood maltreatment experiences and handgrip strength while listening to infant cry sounds. Alyousefi-van Dijk and colleagues posited that greater WM organization might buffer the impact of early life stressors, specifically maltreatment, on aggressive parenting. Further research is warranted to examine early life stress, WM organization, and parenting outcomes in new fathers.

We call for a greater focus on the link between early adversity, parent neurobiology, and parenting outcomes during their infant’s first six months. Research on child behavioral problems suggests that parents’ behaviors as early as the first six months of their infant’s life may predict later childhood competence in socioemotional domains (Guyon-Harris et al., 2023; Dishion and Patterson, 1992; McEachern et al., 2012). Specifically, one study found that parents of six-month-old infants who reported that they enjoyed and supported infants’ positive behavior and participated in proactive parenting were more likely to have children with lower social-emotion problems at six months, higher conceptual vocabulary at 18 months, and higher social–emotional competence at 24 months (Guyon-Harris et al., 2023). Thus, research can further elucidate how parents’ neurobiology may support both early parenting behavior and children’s subsequent long-term development. Given previous research emphasizing that corticolimbic tracts are affected by early adversity (J. T. Condon et al., 2022) as well as potentially supportive of optimal parenting behavior (Feldman et al., 2019), in the current study, we examine associations between parental early adversity, parental neurobiology, and parenting behaviors supporting infants’ socioemotional development.

Using data from a study of first-time fathers transitioning to parenthood, the current study examines associations between expectant fathers’ self-reported exposure to risky family environments, their WM organization during their partner’s pregnancy, and their subsequent parenting effectiveness. A risky family environment is defined as lacking nurturance through aggression, a cold, unaffectionate interaction style, or neglectful behavior (Taylor et al., 2004a). Though risky family environments may have some instances of extreme maltreatment (e.g., physical abuse), the majority of dysfunction in risky family environments is within the range of normative and non-reportable behaviors (e.g., lack of warmth, poorly organized household, use of putdowns). Additionally, as other researchers have noted, to understand how adversity impacts human neurobiology, researchers must examine adversity within community samples that may have experienced adversity on a continuum (Roth et al., 2018).

**Figure 1.**
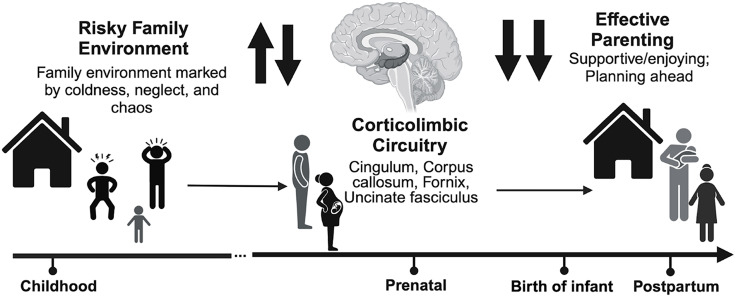
Model positing the link between fathers’ risky family environments on corticolimbic circuitry and effective parenting. *Note.* a conceptual overview of the relationship between risky family environments, corticolimbic circuitry, and effective parenting. Extensive evidence links greater early life stress and lower white matter connectivity in corticolimbic circuitry. The identified white matter circuits (i.e., fornix, cingulum, corpus callosum, and uncinate fasciculus) are highlighted because they connect key corticolimbic gray matter structures (e.g., amygdala, hippocampus, medial prefrontal cortex) involved in emotion regulation. Additionally, the identified white matter circuits are highlighted because existing biobehavioral parenting models emphasize the importance of corticolimbic circuitry for parenting (Feldman et al., 2019). Specifically, we predict higher corticolimbic circuitry supports more effective behaviors relevant to parenting, such as emotion regulation, multitasking, and planning ahead (Condon et al., 2022).

Examining men’s history of risky family environments may provide insight into how suboptimal family dynamics may shape men’s neurobiology and subsequent parenting (Repetti et al., 2002; Taylor et al., 2004a). Until now, no prior research has looked at associations between fathers’ risky family environments, white matter, and parenting outcomes. For our study, we use a model focused on risky family environments linked to brain structure in corticolimbic circuitry involved in executive functioning, such as emotion regulation (Farber et al., 2022; Kopala-Sibley et al., 2020; Figure 1). Importantly, the concept of a risky family environment overlaps with the types of adversities included in the Adverse Childhood Experiences (2020) checklist, a standard screener for the number of adversities an individual has experienced before adulthood. As few studies concentrate on men during the prenatal period, this paper examines prenatal predictors of parenting behavior (Cárdenas et al., 2022). Understanding expectant fathers’ experiences during the prenatal period can clarify whether and how expectant fathers’ baseline neurobiology may support their early caregiving behavior and child development. In addition to being a baseline, this period can allow us to identify the early predictors of those with non-optimal caregiving behavior, which could lay the groundwork for early interventions or risk monitoring.

Previously, we examined how fathers’ WM organization changed across the transition to first-time parenthood, and our group published these findings using a subset of the sample used for this cross-sectional study (Cárdenas et al., 2024). Briefly, we found whole-brain and regional changes in FA from prenatal to postpartum, especially in the cingulum and corpus callosum forceps minor. Postpartum depression symptoms were linked with both increases and decreases in FA, suggesting that the transition to parenthood may be linked with fathers’ neuroplasticity during the transition to parenthood. In the current study, we address the following two aims. First, we aim to identify whether participants’ self-reported risky family environments are associated with their whole-brain WM organization during the prenatal period. We predicted that greater exposure to risky family environments would be related to weaker whole-brain WM organization. Second, we examined associations between risky family environments, whole-brain WM organization, and parenting behavior. We expected that lower FA would be associated with less effective parenting behavior.

## Methods

### Participants

Data for the project came from a neuroimaging sub-study of an IRB-approved longitudinal study examining the transition to first-time parenthood within heterosexual couples. A total of 43 males enrolled in the MRI sub-study. One participant did not complete the scan after enrollment, and a second participant was excluded because of a brain anomaly identified by a board-certified radiologist. Thus, 41 participants had usable brain data collected during the prenatal period. See Table 1 for sample demographics.

**Table 1.** Participant demographics

Variable		Values
Father’s age (years)		31.81 (4.18)
Fetus’ gestational age at scan (months)		6.96 (1.09)
Average amount of time in a typical week the father serves as primary caregiver (hours)	18.8	12.3
Ethnicity (%)	White	13 (31.71 %)
	Black	2 (7.32 %)
	Hispanic or Latino/a	13 (31.71 %)
	Asian or Pacific Islander	10 (24.39 %)
	Multiracial	2 (4.88 %)
Education (%)	High school	1 (2.44 %)
	Some college	7 (17.07 %)
	Associate’s degree	1 (2.44 %)
	Bachelor’s degree	13 (31.71 %)
	Master’s degree	11 (26.83 %)
	Doctoral degree	8 (19.51 %)

### Procedure

Participants in the study completed several visits. First, the expectant fathers and their pregnant partners completed an in-person lab assessment of prenatal experiences at mid-to-late pregnancy and six months of gestation (i.e., *M* = 6.59, SD = 1.12). Roughly two weeks after the prenatal lab visit, the expectant fathers returned for a prenatal scan when their partner was about 6-8 months pregnant (*M* = 6.96, SD = 1.10). When infants were six months old (*M* = 6.52, SD = 0.60), fathers completed questionnaires about their parenting behaviors and the average number of hours in a typical week that the father reported as primary caregiver.

### Neuroimaging acquisition

We collected data using a 3.0 T Siemens MAGNETOM Prisma^fit^ scanner with a 20-channel head coil. Following a T1 scan, DWI images were acquired with the following parameters: 70 contiguous slices were collected in axial orientation; voxel dimension = 2 mm × 2 mm × 2 mm; repetition time, TR = 8100 ms; echo time, TE = 69.0 ms; fat saturation, on; frequency direction, anterior/posterior; field of view = 256 mm × 256 mm; in-plane resolution, 2 mm. One baseline image *b* value of 0s/mm^2^ and 64 different diffusion orientations were acquired with a *b* value of 1000s/mm^2^. The acquisition time for the DWI protocol was 9 minutes and 45 s. Participants were instructed to stay awake and presented with a standard fixation cross throughout the scan.

### Neuroimaging preprocessing

We preprocessed diffusion-weighted images with DTIprep to automatically detect common artifacts, correct for motion and eddy current deformations, and exclude bad gradients. After quality checking, we maintained 41 participants with sufficient gradients (> 18 gradients).

### Neuroimaging preprocessing

Further DWI preprocessing was completed using DSI Studio (http://dsi-studio.labsolver.org/; 11.16.20232). First, the b-table was checked with an automatic quality control procedure by comparing fiber orientations with a population-averaged template (Yeh et al., 2021). Second, the diffusion data were reconstructed in the MNI space using *q*-space diffeomorphic reconstruction (Yeh et al., 2010; Yeh & Tseng, 2011). A diffusion sampling length ratio of 1.25 was used. The output resolution in diffeomorphic reconstruction was 2 mm isotropic. FA was calculated using tensor analysis. Finally, we calculated a goodness-of-fit with an R^2^ statistic between each participant’s quantitative anisotropy and the population-average template. A cutoff of R^2^ > 0.5 was used for quality assurance based on DSI developer recommendations and previous studies (Hodgdon et al., 2022). All participants had R^2^ statistics above 0.71.

### Childhood exposure to risky family environments

The Risky Families Questionnaire (RFQ) was administered at the prenatal visit (Taylor et al., 2004b). The RFQ, an 11-item self-report measure, has a four-point Likert scale (1 = rarely or none of the time; 4 = most or all of the time). This questionnaire was designed to assess the extent to which the childhood family environment (between ages 5 and 15) was cold, conflictual, or chaotic to estimate the degree of risk for physical, mental, and emotional distress participants faced in their homes in childhood and adolescence. Prior research suggests this measure has good construct validity and internal consistency (Coelho et al., 2014; Taylor et al., 2004a, 2006, 2011). In the present study, Cronbach’s alpha was *α* = .86, 95% CI [0.79, 0.90].

### Effective parenting

We administered an adapted version of the Parenting Your Baby (PYB) - 6 months at the postpartum lab visit. Higher scores on the PYB indicate greater endorsement of more effective parenting in the past month (Guyon-Harris et al., 2023). The PYB has two domains of effective parenting: Supporting and Enjoying Your Baby (e.g., “Respond right away when your baby cries or fusses”) and Planning Ahead with Your Baby (e.g., “Distract your baby when s/he was about to get upset?”). The Supporting and Enjoying Your Baby domain can be conceptualized as parental warmth and describes parents’ abilities to delight in a child, feel confident in soothing, and engage in child-led play. The Planning Ahead domain assesses parents’ abilities to anticipate their child’s needs, support exploration, give clear choices, break up tasks, and warn about transitions. Our study used a modified version of the PYB containing 17 items. The additional item was situated in the Planning Ahead Domain compared to the most recent version of the PYB. The additional item was “Think about parenting your baby in the past month. Were you able to point to and name objects and people?” The PYB utilizes a 7-point Likert scale (0 = Not at all; 7 = Most of the time). In the present study, Cronbach’s alpha for the PYB was *α* = .90, 95% CI [0.86, 0.92]. Of note, our paper found that fathers reported a high level of effective parenting (*M* = 6.00, SD = .61), consistent with other papers examining the validity of the PYB (Guyon-Harris et al., 2023).

### Statistical analysis

#### Correlational tractography

To examine our first hypothesis, which was to examine the relationship between risky family environments and WM organization, we used DSI Studio (Yeh et al., 2021) to derive correlational tractography. Specifically, the correlation between FA and risky family environments was computed using a nonparametric Spearman correlation after partially regressing the effect of infant and father age on FA. Deterministic fiber tracking was applied to map correlational tractography with t-statistics greater than 2 and a minimum tract length of 40 mm.

The findings were then examined against a random permutation using the connectometry analysis (Yeh et al., 2016). Specifically, the connectometry analysis applied a permutation test by randomly permuting the subject’s demographics (permutation count = 4,000) to derive a null distribution of the length of correlational tractography. FDR was then computed by comparing the length of the correlational tractography between null results and non-permuted results. An FDR threshold of 0.05 was used to filter the results. This approach is consistent with many existing studies utilizing correlational tractography and connectometry (Hodgdon et al., 2022; Yeh, 2013; Yeh et al., 2019). To examine whether FA values were linked with education level, we planned to extract FA values for each subject and investigate correlations with education level using a Spearman rank-order correlation.

We used Pearson correlations to examine our second hypothesis, assessing the linear relationship between white matter organization associated with risky family environments and effective parenting. Prior research has found that fathers’ neural activation is related to the time spent engaging in child care (Abraham et al., 2014). Additionally, previous studies, using parent and non-parent populations, have found links between white matter microstructure, stressful events, and depression symptoms (Cárdenas et al., 2024; Meng et al., 2021; Silver et al., 2018). Thus, we examined whether these results were impacted by three covariates: average weekly number of hours spent as the primary caregiver, whether their postpartum visit occurred following the pandemic’s start, and fathers’ depression levels at postpartum.

## Results

### Association between risky family environments and prenatal FA

**Figure 2.**
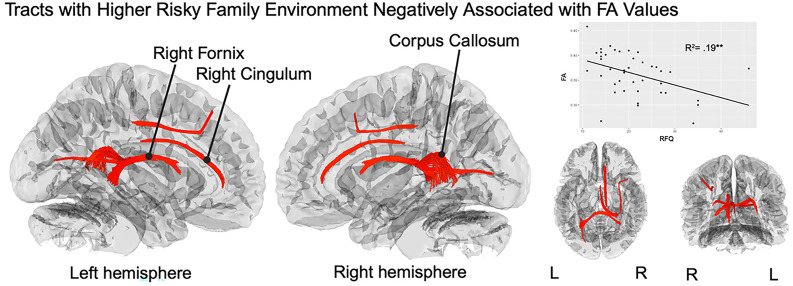
White matter tracts negatively correlated with the risky family questionnaire. *Note.* The figure depicts the results of correlational tractography analyses examining the link between early risky family environments and fractional anisotropy. Analyses control for father age and gestational age of the fetus. The cerebellum was excluded from the analysis, and the seeding region was placed at the whole brain. T-score threshold = 2., FDR < 0.05. Three tracts labeled. RFQ = risky family questionnaire, FA = fractional anisotropy, L = left, R = right. Scatter plot on top right depicts the relationship between RFQ and prenatal FA values of tracts negatively associated with RFQ. R^2^ value is listed. ^*^ *p* < .05, ^**^ *p* < .01.,^***^ *p* < .001.

Connectometry analyses revealed several tracts in which generalized fractional anisotropy (FA) values were negatively correlated with fathers’ reports of a risky family environment in childhood (FDR = 0.000; Figure 2), including the right fornix, corpus callosum tapetum, and right parolfactory cingulum. All tracts are presented in Table 2. Spearman’s rank-order correlation was run to determine the relationship between education levels and fractional anisotropy values extracted from the correlational tractography analysis. A negative correlation between these scores was not statistically significant (*r*(39) = −.021, *p* = .90).

**Table 2.** False discovery rates for negative and positively correlated tract bundles (t-threshold = 2)

Bundle regions	Number of tracts	Mean length (mm)	FDR
Right fornix	261	48.584	0.000
Corpus callosum tapetum	128	44.977	0.000
Right parolfactory cingulum	110	47.462	0.000
Corpus callosum forceps major	45	45.248	0.000
Right superior longitudinal fasciculus	26	44.845	0.000
Right arcuate fasciculus	3	40.662	0.000
Right frontal parietal cingulum	1	41.999	0.000

### Association between FA and effective parenting

**Figure 3.**
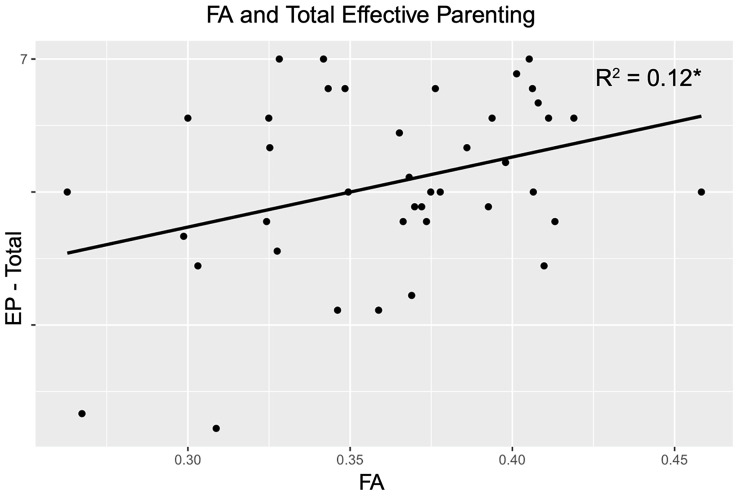
Scatter plot of fractional anisotropy values and total effective p*arenting. Note*. Scatter plot of the Pearson correlations between prenatal fractional anisotropy (FA) values of tracts negatively associated with risky family questionnaire (RFQ) and total effective parenting (EP - total) via the parenting your baby (PYB) at postpartum.

A positive relationship existed between higher prenatal FA values in tracts negatively associated with riskier family environments and the fathers’ total effective parenting behaviors at postpartum (*r*(39) = .37, *p* = .02; Figure 3). Table 3 provides means, standard deviations, and correlations for study variables. These associations were consistent and remained significant when examining the two subscales, Supporting/ Enjoying and Planning Ahead (see Supplemental Material). Additionally, the association between prenatal FA values in tracts negatively associated with riskier family environments and the fathers’ total effective parenting behaviors at postpartum remained significant after running partial correlations controlling for whether fathers provided postpartum data before or after the COVID-19 pandemic, *r*(39) = .38, *p* = .01; the average total number of hours fathers reported as the primary caregiver, *r*(35) = .38, *p* = .02; and postpartum depression symptoms at 6 months postpartum, *r*(39) = .36, *p* = .02.

**Table 3.** Means, standard deviations, and correlation coefficients for study variables

Variable	Mean	SD	Range	1	2	3
1. Risky family	21.10	7.39	11 – 46	1.00		
2. FA	0.36	0.04	0.26 – 0.46	−.44^**^	1.00	
3. Effective parenting	6.00	0.61	3.99 – 7.00	−.22	.37^*^	1.00

*Note.* Risky family environments are measured via the Risky Family Questionnaire (RFQ). Parenting is measured via Parenting Your Baby (PYB). FA = Mean fractional anisotropy of tracts negatively correlated with Risky Family Questionnaire. EP = effective parenting. ^*^ *p* < .05, ^**^ *p* < .01.,^***^ *p* < .001.

## Discussion

Among expectant fathers who were scanned during their partner’s pregnancy, we found that men who reported that their own childhood family environments were characterized by harsh, chaotic, or conflictual interactions showed lower fractional anisotropy (FA) in white matter tract bundles, including the fornix, cingulum, and corpus callosum, indicating less integrity of structural connectivity. Additionally, lower FA in those regions was related to less effective postpartum parenting, such that fathers with lower FA reported less supportive and proactive parenting behaviors. As expected, risky family environments predicted lower FA within corticolimbic circuitry associated with executive functioning and emotion regulation. For example, the cingulum, a tract that connects the limbic lobe and the cingulate gyrus, is frequently identified as a white matter tract shaped by early life stress (Choi et al., 2009; Siehl et al., 2018). The cingulum processes emotion, pain, and memory (Choi et al., 2009). In addition, the corpus callosum is the largest and most expansive white matter tract and often shows alterations associated with childhood-onset PTSD (Siehl et al., 2018). Similarly, alterations in the fornix, which connects the hippocampus to the hypothalamus, are consistently associated with early life risk (Hakamata et al., 2022; Lim et al., 2020; McCarthy-Jones et al., 2018). Given that the corpus callosum, especially the tapetum and fornix, are implicated in the encoding and retrieving of memories, a possible interpretation of our results is that riskier family environments may be related to altered FA in regions that support emotion regulation and episodic memory (Dennis et al., 2021), which in turn may have compromised fathers’ ability to parent effectively.

Though our study focuses on a community sample of first-time fathers, our findings dovetail with research on early life stress and FA in clinical populations. For example, a systematic review of the literature examining FA in adults and children with post-traumatic stress disorder (PTSD) found that both adult PTSD patients with childhood trauma and children diagnosed with PTSD were more likely to show decreased FA within the corpus callosum compared to healthy subjects (Siehl et al., 2018). Furthermore, extensive research in clinical and subclinical samples links lower FA in the cingulum with chronic stress (Zhang et al., 2013). In line with this research, our findings, reflecting fathers reporting a range of risky family environments, revealed an association between risky family environments and lower FA in both the corpus callosum and cingulum.

Our findings build on emerging evidence of FA and effective parenting. We found that lower FA in regions associated with risky family environments was related to less effective parenting, including proactive, planful, supportive, and enjoyable parenting when infants were roughly six months old. Though few studies have focused on FA and parenting, parenting researchers posit that corticolimbic circuits are vital for emotion regulation skills, which are foundational for parenting behavior (Rutherford et al., 2015). Extensive research has linked early life stress, FA, and cognitive functioning. For instance, prior evidence suggests individuals who have experienced early neglect show lower FA in several regions, including the cingulum, and lower FA was related to less optimal cognitive functioning (i.e., spatial planning, visual learning, and memory) (Hanson et al., 2013). Similarly, a review article examining white matter organization and executive functioning found several white matter tract bundles, including the cingulum, fornix, and corpus callosum, associated with various cognitive functioning measures (Ribeiro et al., 2024). Our paper builds on previous clinical and cognitive neuroscience research to provide evidence linking FA and parenting effectiveness.

This study had several strengths. First, this study represents one of the few investigations of the neurobiology of expectant first-time fathers. It can spur greater research on men and fathers within the context of parenting. Second, the study examines early life stress using a measure (i.e., Risky Family Questionnaire) that captures less extreme and more common forms of early life stress. In doing so, the project provides more translatable insight into how less severe forms of early life stress may relate to adult structural connectivity. Third, the study leverages a longitudinal design by collecting neuroimaging data at prenatal and self-reporting parenting behavior at postpartum. As such, the project provides greater insight into predictive neurobiological mechanisms underlying fathers’ adjustment to parenthood.

This study had several limitations. First, given that the scope of our project was to examine less severe forms of early life stress, our study does not examine more aversive forms of early life stress, such as child maltreatment. Given frameworks suggesting that dimensions of early life stress, such as deprivation and threat, may yield distinct effects on neural development (McLaughlin et al., 2014), future studies examining early adversity and father neurobiology would benefit from gathering more precise data on different dimensions of early adversity. Second, our project measured parenting behavior using self-report measures, which are vulnerable to bias. Future studies can benefit from incorporating behavioral coding of father–infant interactions. Third, our sample was racially and ethnically diverse but high in average educational attainment and restricted only to fathers who were cohabiting with their partners, which limits the generalizability of our findings. Finally, our sample size is similar to other studies examining the neurobiology of parenting. However, studies with larger sample sizes are required to ensure the replicability and robustness of our findings.

In conclusion, our findings suggest that risky early family environments are associated with lower FA in expectant fathers and that lower prenatal FA predicts less effective postpartum parenting. Given the importance of the perinatal period for new parents, our findings may help target fathers who struggle with parenting during the transition to fatherhood. These findings are largely consistent with existing research on the neurobiological and psychological consequences of early life stress on corticolimbic circuitry, and they extend this research by focusing on men transitioning to first-time parenthood.
